# Identification and characterization of two novel centriolar appendage component proteins

**DOI:** 10.1186/2046-2530-1-S1-P44

**Published:** 2012-11-16

**Authors:** JM Schrøder, M Rogowski, L Jakobsen, K Vanselow, S Geimer, LB Pedersen, JS Andersen

**Affiliations:** 1 University of Southern Denmark, Denmark; 2 University of Bayreuth, Germany; 3 University of Copenhagen, Denmark

## 

The mother centriole forms the basis of the primary cilium. As the cilium assembles, the mother centriole matures and differentiates into the basal body, and a number of fibrous structures are formed that add to the complexity, including the distal and subdistal appendages. A number of the proteins corresponding to these structures are identified already, but given the complexity of the basal body, the macromolecular composition of some of these appendages remain unknown. To date, the proteins ninein and Cep170 are believed to be a part of the subdistal appendages, and Cep164, outer dense fiber 2, ODF2/cenexin, Cep290, Ofd1 compose the distal appendages. Most of these appendage structures have been reported to play a role for cilia assembly. We previously characterized the centrosome proteome of human lymphoblastic KE-37 cells using quantitative mass spectrometry, which identified 40 novel candidate proteins (Jakobsen et al., 2011). Using immunoflourescence- and immunogold electron microscopy on different human culture cells we identified proteins localizing asymmetrically to the centrioles, and two of these appeared to be new appendage proteins, one distal- (Cep37) and one subdistal (Cep128). Interestingly, in addition Cep37 also localize to the ciliary tip and more faintly along the axoneme. Preliminary results indicate that depletion of Cep37 reduces length of cilia in RPE cells. Cep128 does not affect RPE or hFF cells ability to form primary cilia, but they do show a higher number of pericentriolar dense bodies or satellites as well as a higher number of non exocytotic vesicles in line with the cilium.

